# Efficacy of continuous apical negative ultrasonic irrigation (CANUI) in penetration of simulated lateral canals in extracted teeth

**DOI:** 10.1038/s41598-021-90430-0

**Published:** 2021-05-25

**Authors:** Pablo Castelo-Baz, Francisco J. Rodríguez Lozano, María J. Ginzo-Villamayor, Ramón Miguéns Vila, Juan Seoane-Romero, José Martín-Cruces, Benjamín Martín-Biedma

**Affiliations:** 1 Facultad de Odontología, University of Santiago de Compostela, Entrerríos Street, No. 15702, Santiago de Compostela, Spain; 2 University of Murcia, Murcia, Spain; 3 Universidad Internacional de Catalunya, Barcelona, Spain

**Keywords:** Dental diseases, Root canal treatment, Dentistry, Dental equipment, Dental treatments, Endodontics, Infection control in dentistry, Oral microbiology

## Abstract

The aim of this study was to evaluate the efficacy of continuous apical negative ultrasonic irrigation into simulated lateral canals and the apical third in straight and curved root canals. Two simulated lateral canals were created 2, 4 and 6 mm from the working length in 120 single-rooted teeth (6 canals/tooth, n = 360 straight, n = 360 curved). The teeth were randomly divided into 3 experimental groups: positive pressure irrigation (PPI) (n = 20); passive ultrasonic irrigation (PUI) (n = 20); continuous apical negative ultrasonic irrigation (CANUI) (n = 20). 20% Chinese ink was added to a 5% sodium hypochlorite solution and delivered into the root canals. The results showed a significantly higher (*P* < 0.05) penetration of irrigant into the lateral canals and up to working length in the CANUI group for straight and curved roots. CANUI improves penetration into the lateral canals and up to the working length of the cleared teeth in straight and curved roots.

## Introduction

Apical periodontitis is a biofilm-related infection, and one of the primary goals of treatment is to kill or remove microbes from the root canal system^1^. However, complete elimination is difficult in anatomically complex areas of the root canal system, which are often inaccessible to instruments^2^. Therefore, instrumentation must be combined with adequate irrigation to complete the cleaning process and decrease the microbial load within the root canal system^3^. However, curvatures, which are common in root canals^4^, can reduce the cleaning efficacy of various irrigation techniques^5,6^. Two important factors should be considered during irrigation: (a) whether the system can deliver irrigant to the entire root canal system, particularly the apical third, and (b) whether it is capable of debriding areas not accessible by mechanical instrumentation, such as lateral canals and isthmuses^3,7,8^. Consequently, it is important to investigate whether irrigant systems can introduce irrigant into the apical third of the root canal and into lateral canals.

The most common irrigation method is the conventional type using an irrigating cannula with the front extremity or side coupled to a syringe. However, this method is limited for cleaning the apical portion and areas such as isthmuses and lateral canals^3,9^.

Passive ultrasonic irrigation (PUI), which uses ultrasound to induce cavitation and acoustic waves, is also used to agitate irrigating solutions, thereby improving irrigant properties and cleaning ability in anatomically complex areas^10,11^. Nevertheless, this approach may result in uncontrolled removal of dentin in straight root canals^12^.

To address the limitations of these techniques, several modifications have been proposed. Gutarts et al.^13^ developed continuous ultrasonic irrigation (CUI) using an ultrasonically activated needle placed inside the root canal with a flow of NaOCl, enabling continuous replenishment. Using this method, in vivo studies have shown high cleaning efficiency in areas inaccessible via instrumentation^7,14^. In addition, an in vitro study showed improved penetration of irrigants in lateral canals compared with PUI^15^. However, this approach can transport irrigant beyond the distance at which the instrument acts, leading to possible extrusion of sodium hypochlorite (NaOCl) into the periapical tissues^16^. On the other hand, various studies have supported the safety of negative-pressure cleaning systems compared to irrigation with a syringe or ultrasonic irrigation^17,18^.

**Figure 1:**
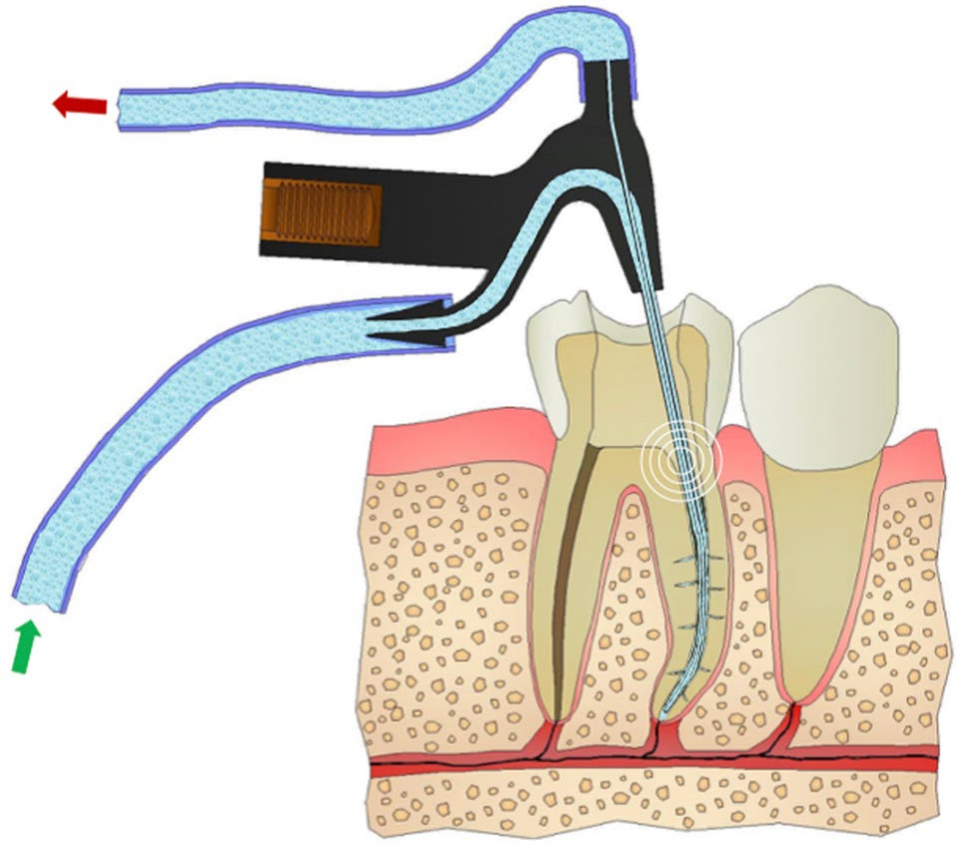
Representation of CANUI design and mode of operation.

**Figure 2:**
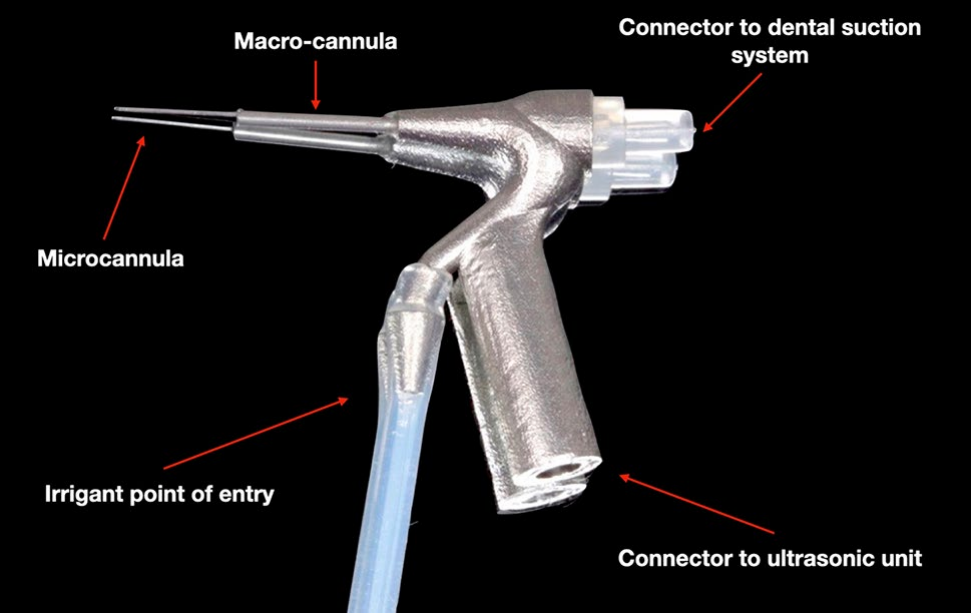
Prototype of CANUI.

**Figure 3:**
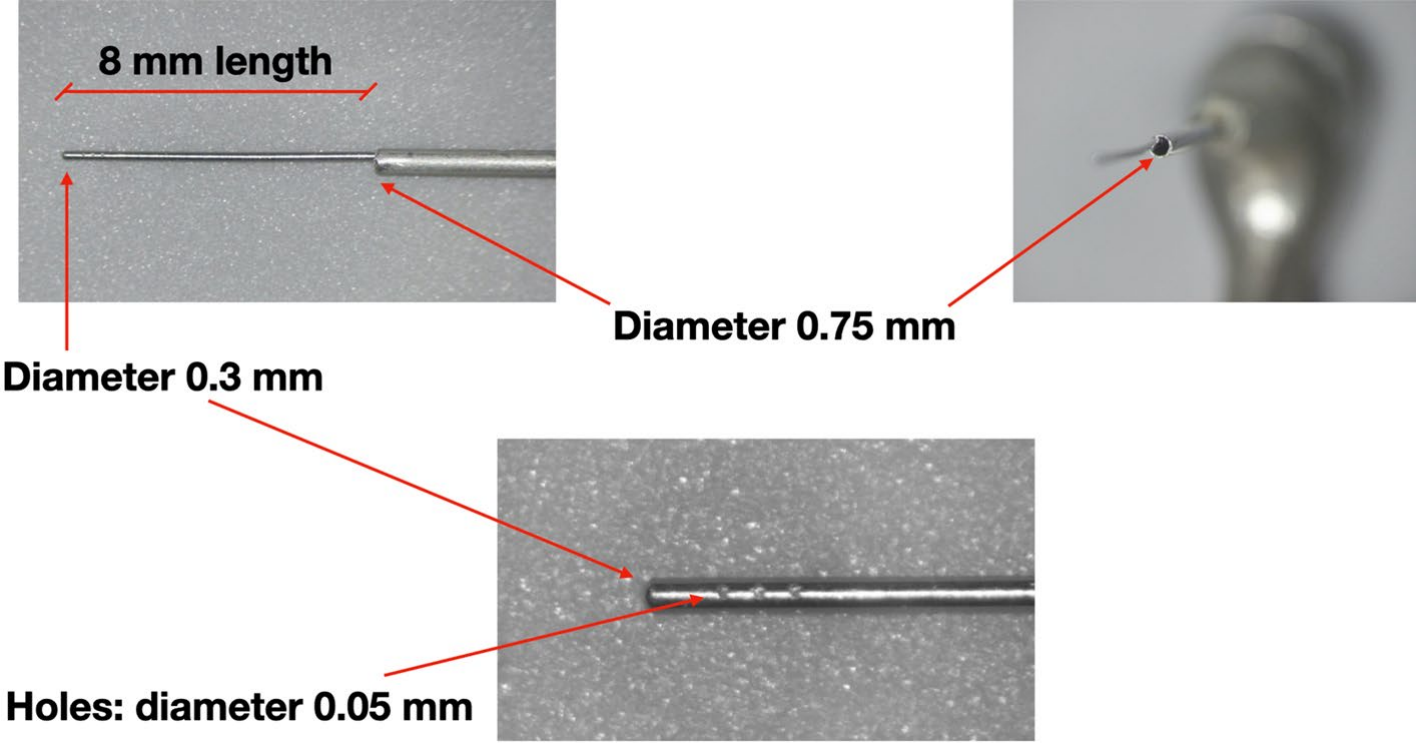
Micro and macro-cannula design and measurements.

**Figure 4:**
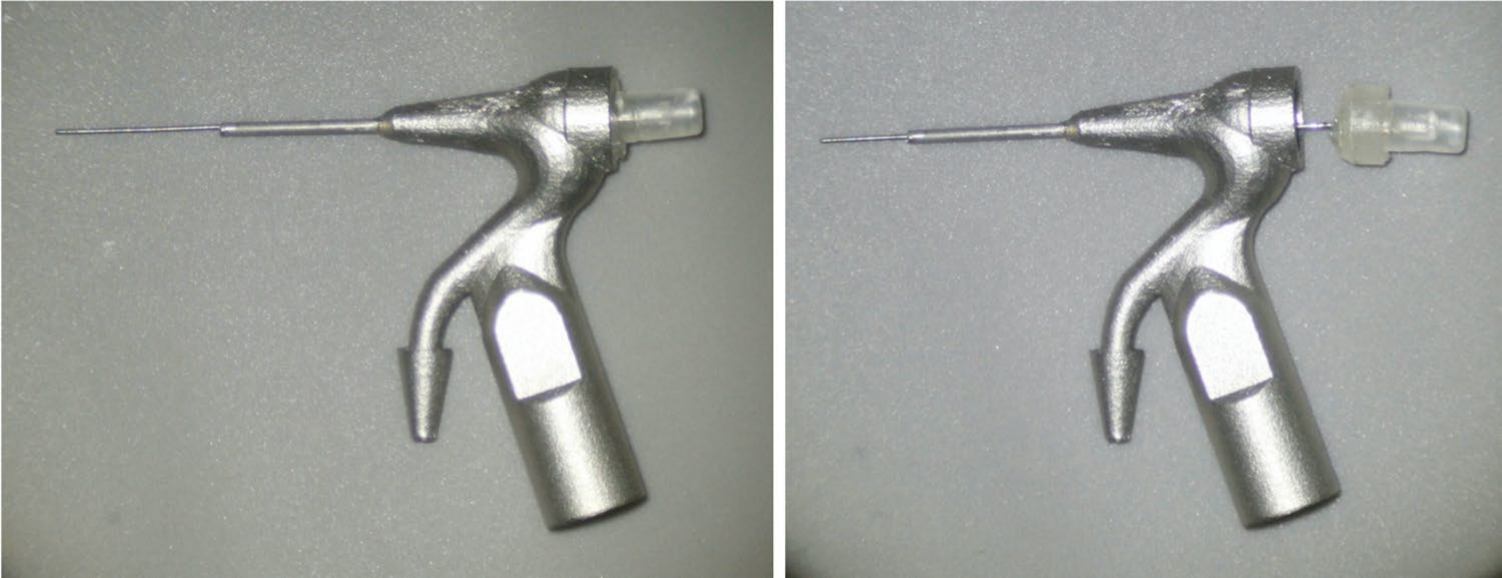
Images showing the assembly of the device (introduction of the micro-cannula into the body and macro-cannula).

In this study, we designed a device for continuous apical negative-pressure ultrasonic irrigation (CANUI)^19^, which combines characteristics of the negative-pressure cleaning systems (to avoid apical extrusion and transport irrigant to the working length) and continuous ultrasonic irrigation systems (to penetrate irrigant into irregularities of the root canal system; Fig. 1). The device consists of a body connected to an ultrasonic unit [Suprasson P5 Booster (Satelec, Acteón Group)] through the lower thread (Fig. 2). From the top this body emerges a steel cannula (that has a pointed diameter of 0.75 mm) and a micro-cannula (0.3 mm) inserted into a cannula (Figs. 3, 4). This micro-cannula is constructed using nickel–titanium and can adapt to the anatomy of curved root canals.

The purpose of this study was to compare the effects of three irrigation systems (PPI, PUI, and CANUI) on irrigant delivery into the apical third of the root canal and into simulated lateral canals in cleared extracted teeth in straight and curved root canals.

## Methodology

### Ethics declarations

This research was carried out in accordance with relevant guidelines and regulations, followed after ethics committee approval (Comité de Ética de Investigación de Galicia, 2016/269). All biological samples were included after obtaining the informed constent from all subjects.

### Methods

120 extracted human single-root teeth with fully formed apices (maxillary central with straight canal and morphology) that had not undergone prior endodontic treatment were selected of these, 60 teeth were straight, and 60 had root curvatures of 20–30º, as determined using the Schneider method^20^. After debriding and complete cleaning of the root surface, samples were immersed in a 5.25% NaOCl (Dentaflux, Madrid, Spain) for 30 min and then stored in saline solution until preparation. The presence of a single canal was verified radiographically and by direct exploration. The same operator performed all experimental procedures. Each specimen was sectioned to a working length of 16 mm. The working length was established under a microscope (M525 F40; Leica, Heerbrugg, Switzerland) at 10× magnification with the tip of #10K-file (Dentsply Maillefer, Ballaigues, Switzerland) visible at the apical foramen. Each root canal was pre-flared using K-Flexofiles (Dentsply Maillefer, Ballaigues, Switzerland) up to #20 and then shaped using ProTaper Next X4 (Dentsply Maillefer, Ballaigues, Switzerland). Irrigation was performed with a 30-G needle (ProRinse; Dentsply Tulsa Dental Specialties, Tulsa, OK) using 3 mL of 5% NaOCl after each file. The irrigation needles were introduced passively up to 2 mm from the working length, and the rate of delivery was fixed at 3 mL/min. The total irrigation time was 10 min/specimen. After instrumentation, all teeth were rinsed for 3 min with 3 mL 17% EDTA (Canal Pro, Coltene Whaledent, Altstätten, Switzerland) followed by a 3-min final rinse with 5% NaOCl. After drying with paper points, the roots were inspected under the microscope at 10X magnification to verify the absence of cracks and to confirm canal cleanliness.

After completing the shaping procedures, teeth were cleared using the modified technique described by Robertson and Leeb^21^ and prepared following the protocol described by de Gregorio et al.^3^. Briefly, teeth were submerged in 5% nitric acid for 36 h, and the solution was renewed every 8 h. Once decalcified, samples were cleared with tap water for 3 min, and lateral canals were created by inserting a 06 C+ file (Dentsply Maillefer, Ballaigues, Switzerland) from the buccal to the lingual wall at 2, 4, and 6 mm from the working length perpendicular to the external surface. Samples were dehydrated in ascending grades of ethyl alcohol and submerged in 99.9% methyl salicylate for clearing and rehardening of dental tissues, as described by de Gregorio et al.^3^. A total of 360 simulated lateral canals were created (6 canals/tooth, 2 lateral canals at each level) for straight canals and 360 for curved canals.

To mimic the clinical situation, a closed system was created by coating each root with soft modeling wax (Cera Reus SA, Reus, Spain). This coating sealed the apical foramen and lateral canals at all three levels. During this procedure, a gutta-percha point was introduced into the canal to the working length to prevent penetration of wax into the canal space.

### Contrast solution

A contrast solution containing 5% NaOCl (80%) and 20% Chinese ink (Sanford Rotring GmbH, Hamburg, Germany) was prepared and delivered to the prepared root canals.

### Experimental groups

**Figure 5:**
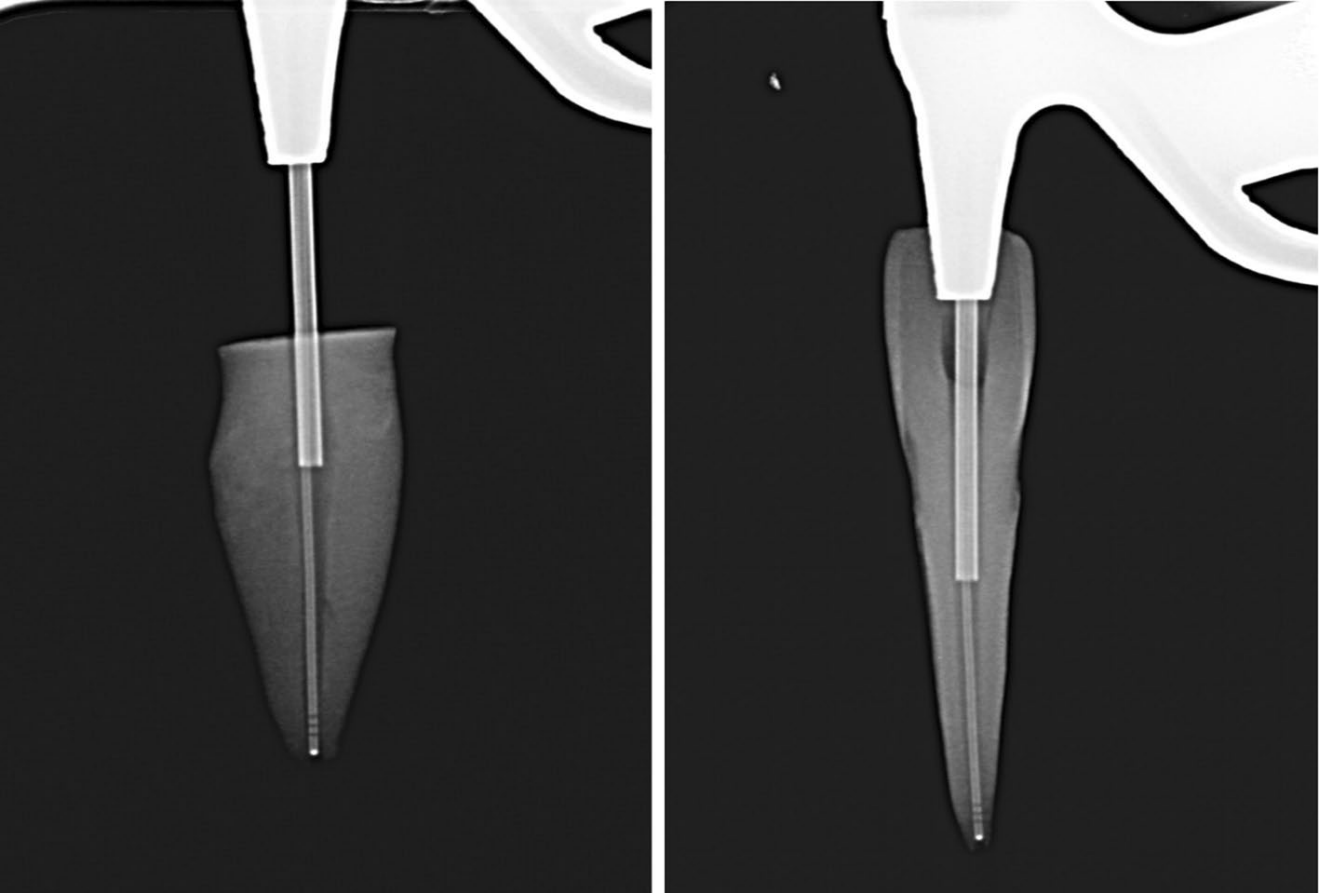
X-rays showing the device in the root canal and the micro-cannula reaching working length (showing both decoronated and non-decoronated roots).

Irrigation was performed in the PPI and PUI groups as described by de Gregorio et al.^3^, with minor modifications. Briefly, irrigation was performed in the CANUI group by introducing the micro-cannula to the working length (Fig. 5). Irrigation time was the same for all three groups (1 min). All procedures were recorded under a dental operating microscope. Details of the irrigation sequences are presented later.

Group 1 (n = 20): Positive pressure (control). Teeth in group 1 were irrigated with PPI (1 min) using a 30-G ProRinse needle and a syringe to 2 mm from the working length. A total volume of 6 mL of contrast solution was delivered. The solution was not dynamically activated in this group.

Group 2 (n = 20): PUI. A total volume of 2 mL of contrast solution was delivered into the teeth in group 2 using a 30-G ProRinse needle, and the solution was left in the root canals. Ultrasonic activation was performed with an ISO15 ESI file (EMS, Nyon, Switzerland) mounted on an ultrasonic unit. The file was passively inserted to 1 mm from the working length and activated using a power setting of 4, as recommended by the manufacturer. The procedure was repeated three times, for a total volume of 6 mL of contrast solution and a total activation time of 1 min for each tooth.

Group 3 (n = 20): CANUI. Continuous apical negative ultrasonic irrigation was performed using prototypes (Figs. 2, 3, 4) of the device mounted on a Suprasson P5 Booster ultrasonic unit. A 10-mL syringe containing contrast solution was attached to the luer-lock connection on the UI needle and inserted to the working length with the microcannula (Fig. 5). Then, the device was activated with the power set to level 6, and the solution was delivered, maintaining a continuous irrigation flow of 6 mL/min. The total activation time was 1 min; a total volume of 6 mL of contrast solution was delivered.

### Evaluation criteria

**Figure 6:**
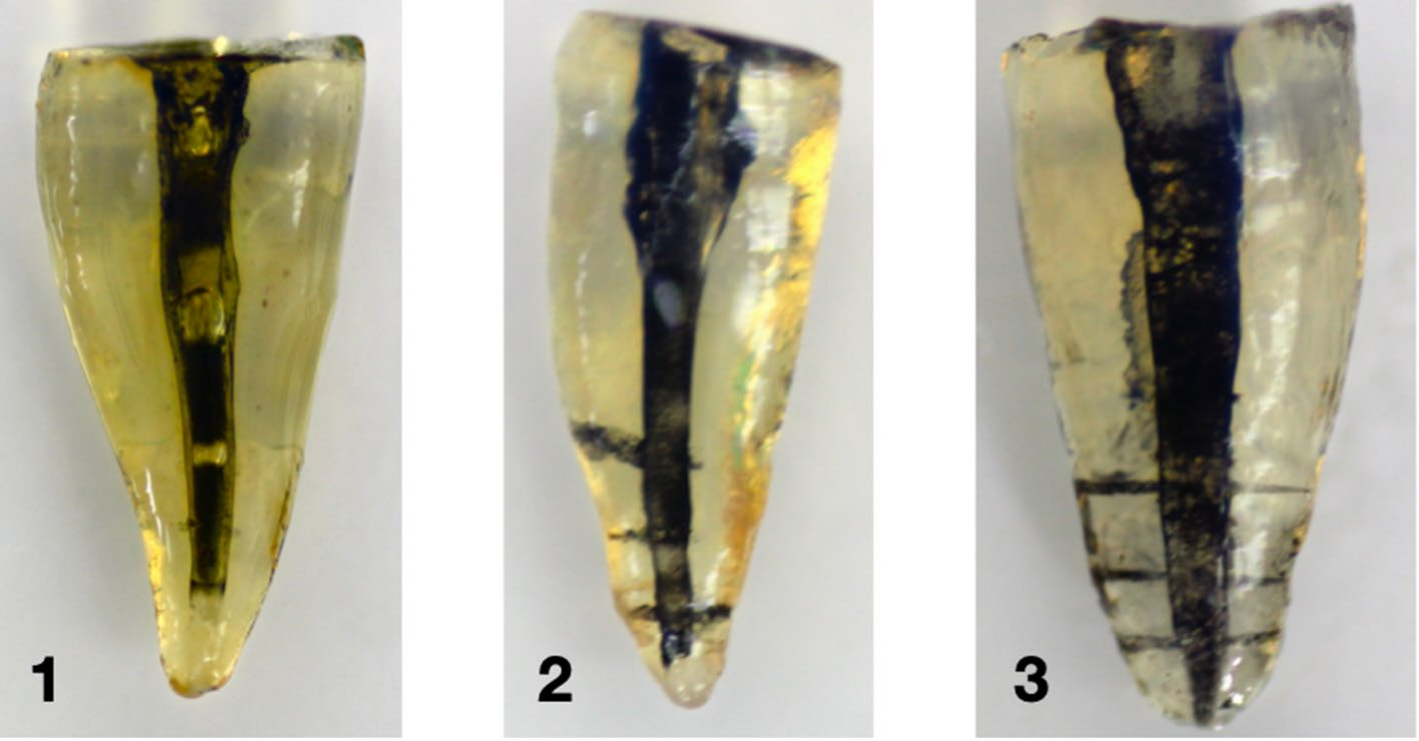
Representative images of straight canals. 1 PPI, 2 PUI, 3 CANUI.

**Figure 7:**
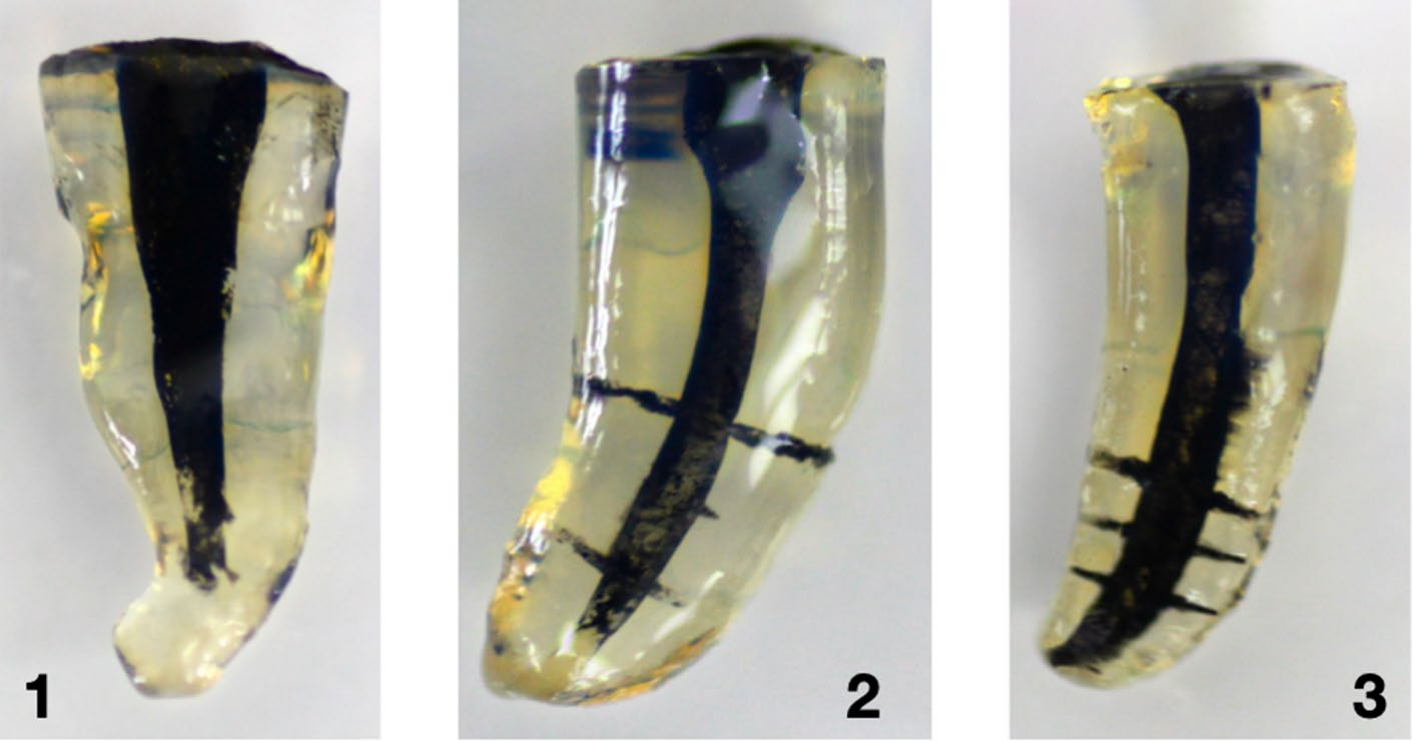
Representative images of curved canals. 1 PPI, 2 PUI, 3 CANUI.

The criteria described by de Gregorio et al.^3^ were used in this study. Samples were evaluated by direct observation of images recorded under the dental operating microscope (Figs. 6, 7). The orientation of all samples was standardized in relation to the recording microscope to produce similar images for all groups.

**Figure 8:**
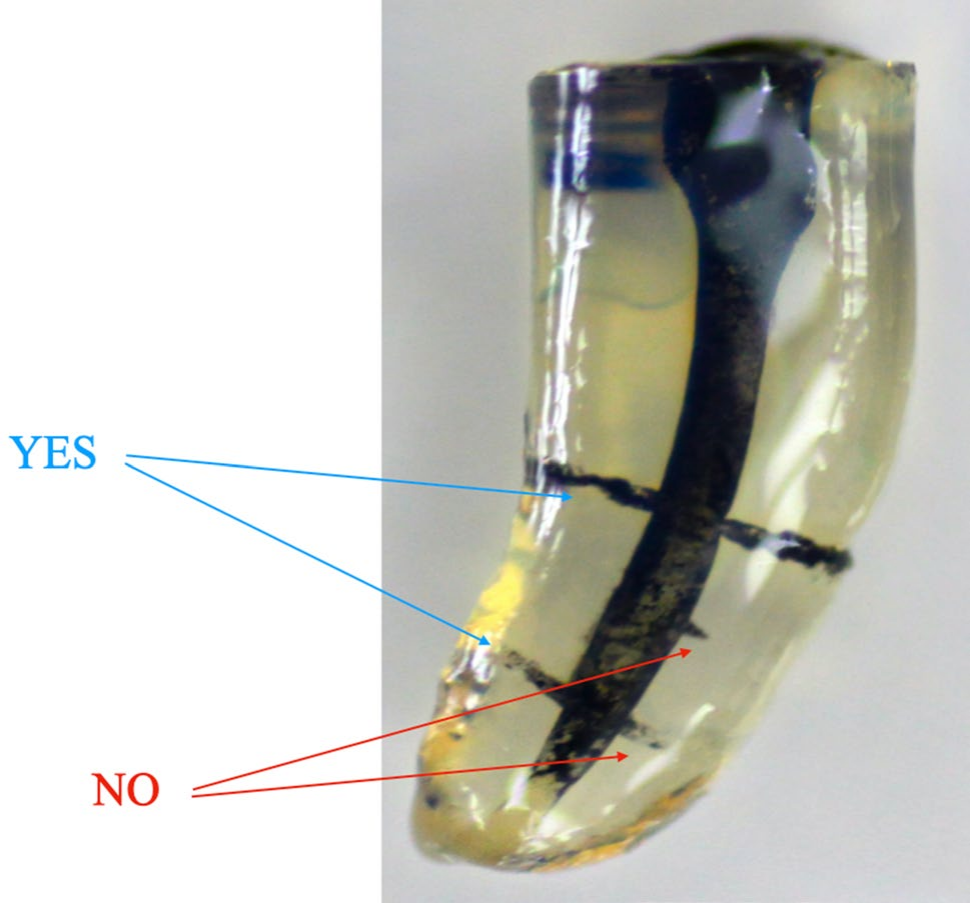
Evaluation criteria: Contrast solution penetrated to at least 50% of the total length.

The penetration of contrast solution into the simulated lateral canals was scored by counting the number of lateral canals (0–2) penetrated to at least 50% of the total length (Fig. 8). The outcome was assessed in each tooth at the three working lengths (2, 4, and 6 mm). One trained evaluator, who was blinded to the group assignment of each sample, scored all samples.

### Statistical analysis

The Mann–Whitney U-test was used to analyze and compare irrigant penetration to the working length and into lateral canals. A comparison between straight and curved canals of root canals was made using the same test. *P*-values of 0.05 were considered statistically significant.

## Results

The flow of irrigant into lateral canals and to the working length was analyzed for all samples (n = 20) in each group in straight and curved canals.

### Reaching the working length

Straight canals: In the first group (PPI, control), the contrast solution did not reach the working length in any sample (0%). The contrast solution reached the working length in 65% of samples in group 2 (PUI) and 100% of samples in group 3 (CANUI). Penetration of the irrigant to the working length was significantly different among the three groups (Table 1).

**Table 1:** Total and percentage of lateral canals penetrated by the irrigation solution in straight root canals.

	Group 1: PPI	Group 2: PUI	Group 3: CANUI	P
Reached working length, n (%)	0 (0)	13 (65%)	20 (100%)	< 0.001
Overall	0 (0)	40 (33.33%)	104 (86.67%)	< 0.001
6 mm	0 (0)	15 (37.5%)	38 (95%)	< 0.001
4 mm	0 (0)	10 (25%)	35 (87.5%)	< 0.001
2 mm	0 (0)	15 (37.5%)	31 (77.5%)	< 0.001
No. of canals penetrated, mean	0	0.33	0.87	< 0.001
% total canals penetrated	0	33.33%	86.67%	
Standard deviation	0	0.47	0,34	

Curved canals: Penetration was complete in 0% of group 1, 35% of group 2, and 100% of group 3 (CANUI). Penetration of the irrigant to the working length differed significantly among the three groups (Table 2).

**Table 2:** Total and percentage of lateral canals penetrated by the irrigation solution in curved root canals.

	Group 1: PPI	Group 2: PUI	Group 3: CANUI	*P*
Reached working length, n (%)	0 (0)	7 (35%)	20 (100%)	< 0.001
Overall	0 (0)	43 (35.83%)	98 (81.67%)	< 0.001
6 mm	0 (0)	20 (50%)	37 (92.5%)	< 0.001
4 mm	0 (0)	13 (32.5%)	35 (87.5%)	< 0.001
2 mm	0 (0)	10 (25%)	26 (65%)	< 0.001
No. of canals penetrated, mean	0	0.36	0.82	< 0.001
% total canals penetrated	0	35.83%	81.67%	
Standard deviation	0	0.82	0.39	

When we compared curved and straight canals, we found no significant difference, although the penetration was greater in straight canals for PUI.

### Penetration into lateral canals

**Figure 9:**
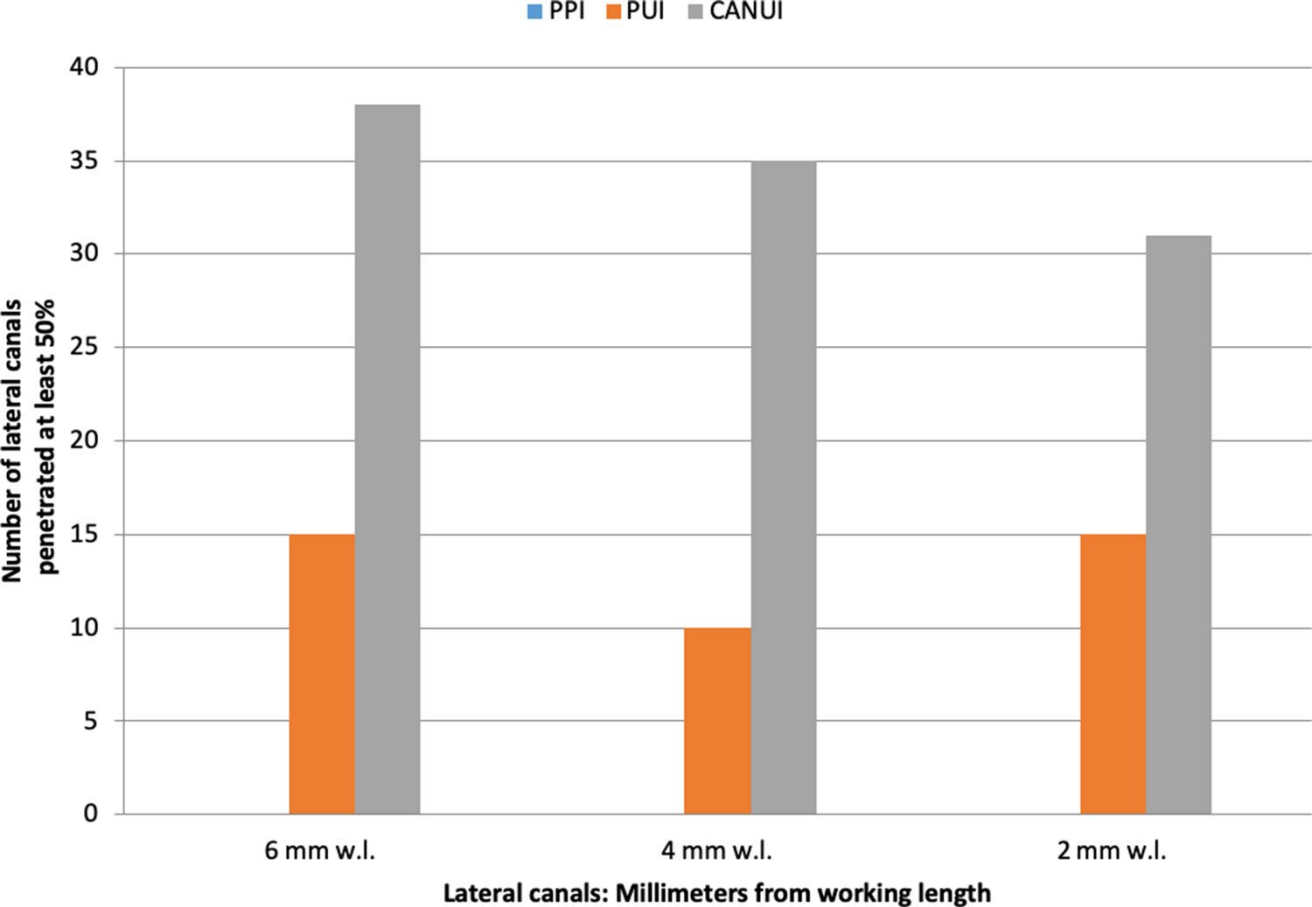
Graphical representation of straight root canals penetrated by the irrigation solution.

Straight canals: Overall penetration into lateral canals was 0% for group 1 (PPI), 33.33% for group 2 (PUI), and 86.67% for group 3 (CANUI) (Figs. 6, 9). These values differed significantly among the groups (*P* < 0.001). These results were confirmed in separate analyses of the three levels (Table 1).

**Figure 10:**
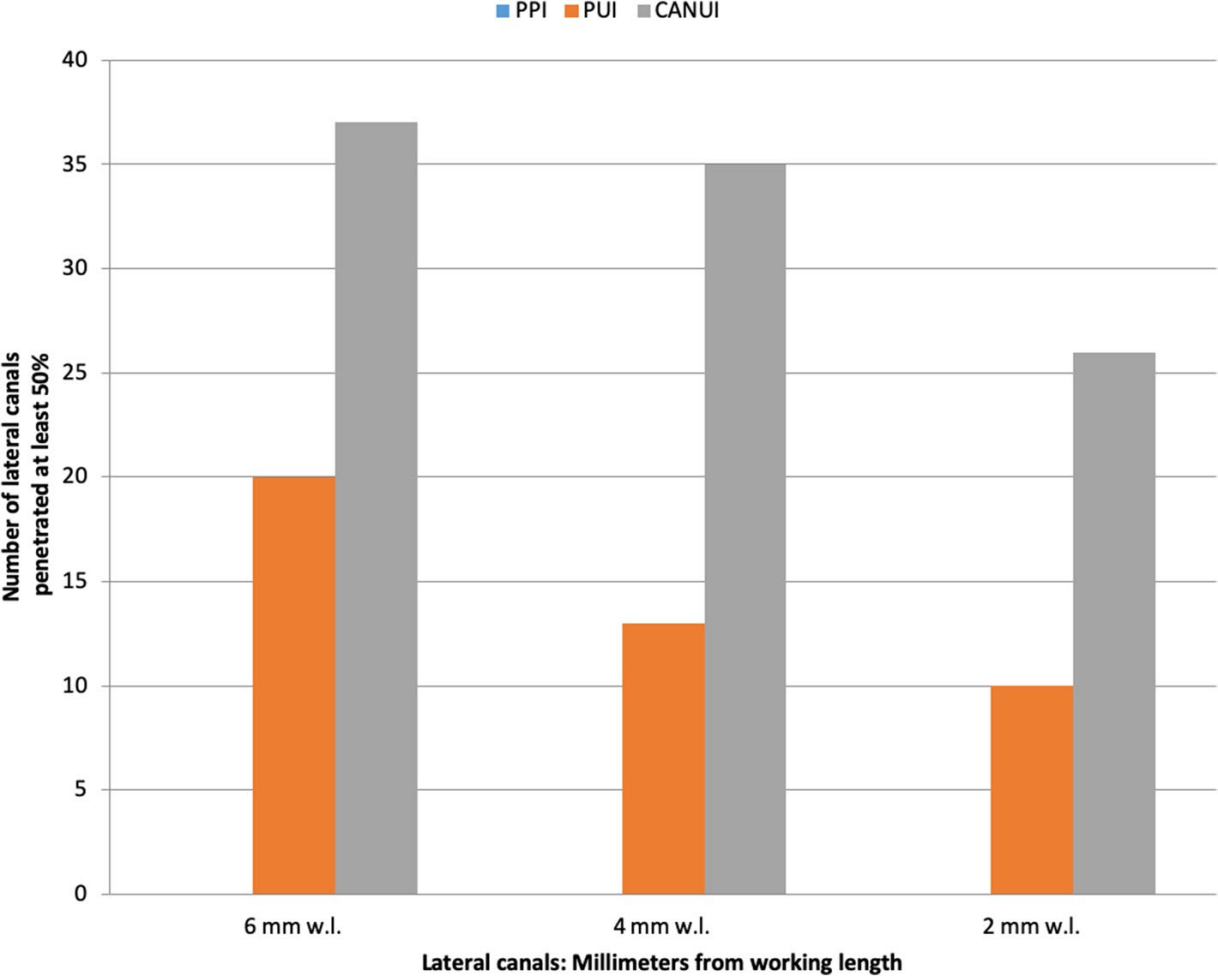
Graphical representation of curved root canals penetrated by the irrigation solution.

Curved canals: Overall penetration into lateral canals was 0% for group 1 (PPI), 35.83% for group 2 (PUI), and 81.67% for group 3 (CANUI) (Figs. 7, 10). These values differed significantly among the three groups (*P* < 0.001). These results were confirmed in separate analyses for the three levels (Table 2).

There was no significant difference between straight and curved canals in terms of PUI penetration into the lateral canals; similar results were found in the CUI group.

## Discussion

The purpose of chemo–mechanical root canal preparation is to clean, disinfect and create a space in the root canal system for the subsequent filling procedure^22^. The goal is to clean the canal system before sealing to prevent the microorganism survival, reinfection, and subsequent inflammatory host reactions^23^. Lack of healing is often attributed to persistent intra-radicular infection in previously uninstrumented canals, dentinal tubules, or in the complex irregularities of the root canal system^24,25^. To avoid infection and develop a safe device, a new device, called CANUI, was designed that integrates advantages of continuous ultrasonic irrigation (CUI) and apical negative pressure (ANP). This system allows simultaneous continuous irrigant delivery and continuous ultrasonic activation, which is possible because the device is connected directly to the ultrasound unit, provides continuous replenishment of the solution (crucial for organic tissue dissolution^26^), and is activated without direct contact between the ultrasonic file and solution.

Based on a previous report, it is important to standardize specimens to avoid anatomic biases that may interfere with the outcomes. Therefore, despite natural variations in teeth morphology, attempts were made to ensure comparability of the groups in root canal morphology. In this in vitro investigation, we compared the ability of two ultrasonic irrigation techniques (PUI and CANUI) and one traditional technique (PPI) to deliver a contrast dye-containing irrigating solution into the apical third of straight and curved root canals and into artificially created lateral canals in cleared extracted teeth. To reproduce the clinical situation, we used an in vitro closed-end canal design that closely replicates in vivo scenarios^27,28^, in which the apical foramen is enclosed in alveolar bone and the periodontal ligament^7,13^. Clinically, this design forces the irrigants to exit the canal coronally rather than apically or laterally^3^. As shown in previous reports^3,15^, irrigation with PPI did not reach the working length or penetrate lateral canals. This may be due to the presence of an apical vapor lock created by the organic decomposition of NaOCl into a bubble of carbon dioxide and ammonium^29^. Furthermore, PPI does not have enough force to penetrate lateral canals in either straight or curved canals.

We also compared two ultrasonic techniques (PUI and CANUI) with each other and with PPI. Both ultrasonic techniques produced adequate irrigant penetration into the apical third of the root canal, but CANUI reached the apical third in all cases, demonstrating significantly greater success than the other groups. This may be due to the apical force of suction of ANP, as demonstrated by de Gregorio et al.^3^. This can be explained by the design of the microcannula, which eliminates vapor lock and allows for apical exchange of irrigants. On the other hand, the efficacy of PUI depends on penetration of the ultrasonic file to 1 to 2 mm from the working length, and the volume of activated solution is limited^30^. We found no statistical difference between straight and curved canals.

Finally, it was compared the penetration of irrigant into the simulated lateral canals; significant differences were observed among the three groups (*p* < 0.05). Previous studies have observed greater efficacy of PUI when compared syringe and needle (PPI) and other techniques^31,32^. The effectiveness of this method for activating irrigants may be due to the production of acoustic microwaves and cavitation^33^. However, some studies have contested its efficacy in curved canals^7,34^, possibly because the ultrasonic tip should not act freely inside the canal space and may bind to the walls and interfere with acoustic streaming and cavitation, resulting in a poorer debridement^35^. In our study, there was no significant difference between straight and curved canals in PUI groups, although we observed increased penetration into the apical third of the straight canals. Moreover, complications such as ledge formation, uncontrolled cutting of the root canal walls, and perforations have been reported using PUI^35^. In the present study, we applied ESI instruments made of nickel titanium for increased flexibility, which may improve the quality of the instrument compared to steel, to avoid these problems. In this investigation, we didn´t observe these complications. On the other hand, CANUI groups showed improved penetration into lateral canals compared with PUI in curved and straight canals. This may be due to the continuous exchange of solution and the optimized activation of the solution as it passes through the ultrasonically energized needle, enhanced by the force of the ANP.

Limitations of this study include the lack of similar studies for comparison. It can be concluded that under the conditions of this study, CANUI improves penetration into the lateral canals and up to the working length of the cleared teeth in straight and curved roots.
